# Dynamics of cerebral blood volume during and after middle cerebral artery occlusion in rats – Comparison between ultrafast ultrasound and dynamic susceptibility contrast-enhanced MRI measurements

**DOI:** 10.1177/0271678X231220698

**Published:** 2023-12-21

**Authors:** Bart AA Franx, Florent Lebrun, Lois Chin Joe Kie, Thomas Deffieux, Denis Vivien, Thomas Bonnard, Rick M Dijkhuizen, CONTRAST consortium, Cyrille Orset

**Affiliations:** 1 Translational Neuroimaging Group, Center for Image Sciences, University Medical Center Utrecht and Utrecht University, Utrecht, The Netherlands; 2 Normandie University, UNICAEN, INSERM UMR-S U1237, Physiopathology and Imaging of Neurological Disorders (PhIND), Institute Blood and Brain @ Caen-Normandie (BB@C), Caen, France; 3 ETAP-Lab, STROK@LLIANCE, 13 Rue du bois de la champelle, 54500, Vandoeuvre-les-Nancy, France; 4 Institute of Physics for Medicine Paris, INSERM U1273, ESPCI Paris, CNRS UMR 8063, PSL Université Recherche, Paris, France; 5 CHU Caen, Department of Clinical Research, CHU Caen, Côte de Nacre, France

**Keywords:** Cerebral blood volume, ischemic stroke, magnetic resonance imaging, reperfusion, ultrafast ultrasound

## Abstract

Tomographic perfusion imaging techniques are integral to translational stroke research paradigms that advance our understanding of the disease. Functional ultrasound (fUS) is an emerging technique that informs on cerebral blood volume (CBV) through ultrasensitive Doppler and flow velocity (CBFv) through ultrafast localization microscopy. It is not known how experimental results compare with a classical CBV-probing technique such as dynamic susceptibility contrast-enhanced perfusion MRI (DSC-MRI). To that end, we assessed hemodynamics based on uUS (n = 6) or DSC-MRI (n = 7) before, during and up to three hours after 90-minute filament-induced middle cerebral artery occlusion (MCAO) in rats. Recanalization was followed by a brief hyperperfusion response, after which CBV and CBFv temporarily normalized but progressively declined after one hour in the lesion territory. DSC-MRI data corroborated the incomplete restoration of CBV after recanalization, which may have been caused by the free-breathing anesthetic regimen. During occlusion, MCAO-induced hypoperfusion was more discrepant between either technique, likely attributable to artefactual signal mechanisms related to slow flow, and processing algorithms employed for either technique. In vivo uUS- and DSC-MRI-derived measures of CBV enable serial whole-brain assessment of post-stroke hemodynamics, but readouts from both techniques need to be interpreted cautiously in situations of very low blood flow.

## Introduction

Perfusion imaging techniques are instrumental in clinical acute ischemic stroke (AIS) diagnosis, patient selection in clinical trials,^1,2^ and translational stroke research. Tomographic perfusion imaging allows in vivo spatial measurement of hemodynamic indices during ischemia and after reperfusion. Hemodynamic parameters, such as cerebral blood flow (CBF) and volume (CBV), can differentially and complementarily report tissue perfusion and may predict subsequent tissue outcome. While CBF has historically enjoyed most scientific interest, there are compelling reasons to study CBV. During occlusion, blood vessels in and around the lesion core dilate, increasing CBV to maintain adequate levels of CBF. High levels of CBV during AIS are thought to reflect good collaterals and associate with good outcome,^3–5^ whereas extremely low CBV in the hyperacute phase may indicate microvascular collapse.^6^ However, extended periods of CBV overshoot have also been observed after the acute phase, spatially coinciding with areas of cellular damage.^7^

CBV can be quantified from the passage of an exogenous contrast agent using magnetic resonance imaging (MRI) through dynamic susceptibility contrast-enhanced perfusion imaging (DSC-MRI).^8^ It relies on fast serial imaging of the passage of an intravenously injected superparamagnetic contrast agent, such as gadolinium DTPA (Gd-DTPA), using high-speed T_2_- or T_2_*-weighted MRI. A characteristic concentration-time curve can be derived from the first passage, from which CBF, CBV and other hemodynamic indices can be calculated.^9^

Ultrafast ultrasound (uUS) is an emerging ultrasensitive Doppler technique used to measure CBV dynamics in experimental neuroscience.^10^ The contrast mechanism relies on flowing red blood cells which backscatter the ultrasonic waves (i.e., echoes), whose time-averaged energy are proportional to red blood cell content, relating the ultrasensitive Doppler signal to CBV.^10^ The uUS application leverages modern electronics to acquire ultrasound images at high frame rates (>10,000 Hz) to a 100 µm in-plane resolution.^10^ Transcranial uUS has been successfully employed to map functional connectivity in rats,^11^ and to monitor cortical perfusion in rat and mouse models of stroke.^12,13^ In addition, uUS experiments can be augmented with a clinically approved microbubble contrast agent, enabling ultrasound localization microscopy (ULM), which improves the signal-to-noise ratio to achieve higher spatial resolutions (∼10 µm in-plane) and allows cerebral blood flow velocity (CBFv) mapping.^14^

For applications in rodents, key differences can be observed between DSC-MRI and uUS setups to measure CBV. Relative to uUS, DSC-MRI requires costly equipment and personnel and yields one hemodynamic readout per certain amount of time at modest spatial resolutions. Yet a major strength is its clinical application. uUS yields continuous high spatiotemporal readouts of CBV, but it requires invasive procedures to mitigate signal attenuation by the skull in rats, ranging from continuous microbubble contrast agent injection^15^ to skull thinning^11,16^ and craniotomy.^17^ Clearly, uUS and DSC-MRI have their utility in translational stroke research, but to date there have been no experiments comparing CBV readouts obtained by both techniques. To that end, we characterized and assessed uUS- and DSC-MRI-derived CBV dynamics during ischemia and subsequent reperfusion in a rat model of filament-induced transient middle cerebral artery occlusion (tMCAO). Additionally, we leveraged the high temporal resolution (0.5 Hz) to measure acute blood flow reinstatement in the brain as the filament was being retracted.

## Materials and methods

Ultrafast ultrasound experiments were performed in France and in accordance with French ethical laws and approved by the local ethical committee of Normandy (CENOMEXA, APAFIS#31474). DSC-MRI experiments were performed in the Netherlands and approved by the Animal Experiments Committee of the University Medical Center Utrecht and Utrecht University. All procedures were in accordance with European Communities Council Directive. Experiments are reported according to ARRIVE guidelines. Data can be made available upon reasonable request.

### Animals

**Figure 1.**
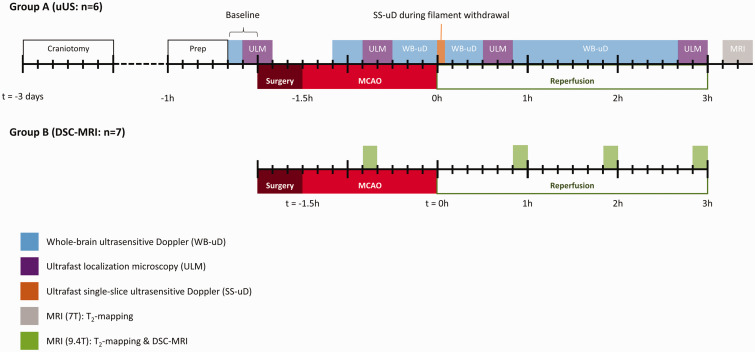
Schematic representation of the experimental workflow per group on a timeline. Blocks on the timeline indicate experiment and their duration. Ultrafast localization microscopy (ULM) (purple blocks) included a 20-minute washout period where no measurements were performed. Large ticks represent hour intervals; small ticks represent 10-minute intervals. WB-uD: whole-brain ultrasensitive Doppler; SS-uD: single-slice ultrasensitive Doppler: ULM: ultrafast localization microscopy; MRI: magnetic resonance imaging; MCAO: middle cerebral artery occlusion.

Experiments were performed on male adult Sprague-Dawley rats (N = 19, 11–13 weeks, Charles-River). Rats were housed under standard conditions, conforming to local legislation, with a light/dark cycle of 12/12 hours and free access to food and water. Since Group A and Group B were studied in two different labs, subjects could not be randomized to either uUS (Group A: n = 6) or MRI experiments (Group B: n = 7). Experimenters could not be blinded to two different imaging techniques. The experimental workflows for both groups are depicted in Figure 1. Measurements were performed at the same time of day for each animal. One animal was excluded (at the site in the Netherlands) because vasogenic edema was not detected in anatomical areas fed by the MCA. Five other animals were excluded due to microsurgery complications resulting in subarachnoid hemorrhage (two in France and one in the Netherlands). One animal died during craniotomy surgery at the site in France. In total, thirteen animals were included for final analysis. There was no premature mortality.

### Anesthesia and monitoring

Before microsurgery and imaging experiments, anesthesia was induced with 4% isoflurane in N_2_O:O_2_ (3:2). Anesthesia was maintained with 2–2.5% isoflurane during microsurgeries, and 1.5% during imaging sessions, while breathing was always unassisted. Rats were restrained in a stereotaxic frame during uUS imaging or in an MR-compatible stereotaxic cradle during MRI experiments. Rats received ophthalmic eye cream (Duratears™ Z, Alcon, Switzerland) before microsurgery and imaging. Animals also received 10 mg/kg subcutaneous lidocaine (Xylocaine 5%, AstraZeneca, Sweden) under the scalp prior to craniotomy, and in the throat area prior to MCAO microsurgery. When anesthetized, body temperature was maintained at 37 ± 0.5 °C by a feedback-controlled heating pad. Blood oxygen saturation and heart rate were continuously monitored throughout surgeries and imaging sessions. To maintain body hydration, 0.9% NaCl was injected after microsurgery and after every hour of imaging to guard hydration status.

### Experimental workflow: craniotomy

To enable assessment of perfusion in the entire brain, including deep structures, we performed a partial craniotomy over the MCA-irrigated area of the brain. Buprenorphine (0.3 mg/kg) was subcutaneously injected 30 minutes before microsurgery began (repeated every twelve hours up until tMCAO). The head was fixed in a stereotaxic frame, the scalp was shaved, and the skin was antiseptically treated with 70% alcohol (Cooper, France) and 10% povidone-iodine (Betadine, Viatris, PA). A sagittal incision was made, and the periosteum was removed to expose the bregma. Using a micro drill, a rectangular shape was drilled around bregma: the length of the rectangle extended along the coronal suture up until the parietal bone, and the width extended approximately ±3 mm along the sagittal suture (with bregma at the center). The bone was drilled away slowly and frequently cooled by cold or room temperature saline, depending on the distance from the dura mater. When the layers of the skull were sufficiently drilled away, the rectangular jigsaw piece of bone could be removed with a forceps while gently separating it from the dura with a blunted needle. This method, instead of slowly drilling away the entire area layer by layer, was chosen to reduce heat trauma to the brain and to repurpose the craniotomized roof of the skull as protection for the now exposed brain until the ischemia-reperfusion experiment. When the slab of skull roof was removed, the exposed dura was soaked with artificial cerebrospinal fluid, and the bone was reattached with dental cement (Ionoseal, Voco, Germany), which was left to harden under LED light for 5 minutes. Finally, the skin was sutured up, povidone-iodine was reapplied, and the rat recovered in its home cage for three days. The procedure took about 45 minutes.

### Experimental workflow: ischemic stroke model

tMCAO was induced as described by Zea Longa et al.^18^ In brief, the right common carotid artery (CCA), internal carotid artery (ICA) and external carotid artery (ECA) were exposed and dissected. The CCA was temporarily ligated while the ECA was ligated and arteriotomized. The ECA was reflected along the ICA such that a silicon-tipped nylon filament (4-0, Doccol Corporation, USA) could be advanced by 22 mm or until resistance was felt. The throat area was sutured up with the uncoated nylon end left protruding out from the skin, such that it could be removed by gently pulling on the nylon 90 minutes after occlusion. This could be achieved in the stereotaxic frame while acquiring data during uUS (Group A), but not in-bore during MRI (Group B).

### Ultrafast ultrasound imaging (group A only)

The timing of the ultrafast ultrasound imaging experiments in Group A are depicted in Figure 1. Before ultrasound imaging, the tail vein was catheterized with a 26 G needle (Abbocath) to inject microbubbles for ULM experiments (see below). Next, the animal was fixated in a stereotaxic frame and lidocaine was reinjected subcutaeously under the scalp. The sagittal incision was reopened and the rectangular bone flap, fixed by dental cement, was gently removed. Acoustic gel (Unigel Bleu, Asept Inmed, France) was applied on the exposed skull and the ultrasound probe was centered above the craniectomized area.

Acquisitions were performed on a functional ultrasound prototype platform (Iconeus, Paris, France & ART Inserm U1273) based on a ultrafast research scanner (128 channels, 62.5 MHz sampling rate, Verasonics) and driving a 15 MHz ultrasound probe (14 MHz, 0.11 mm pitch, 128 elements, 14 mm width) mounted on a motorized stage (Physik Instrumente, Germany) allowing movement with four degrees-of-freedom (3-dimensional translation and rotation along the vertical axis).

For additional details on the motorized setup and coordinate system the reader is referred to earlier work.^12^

#### Ultrasensitive Doppler

200 compounded frames (11 incremental angles between ±10°) were acquired at 500 Hz. To separate signal from the blood from physiological noise, a clutter filter was applied by singular value decomposition, where the first 60 singular values were removed prior to frame summation. During whole-brain ultrasensitive Doppler acquisitions, the motorized stage moved the ultrasound probe in 21 incremental steps of 0.3 mm posterior-to-anterior, with a 1.2 second pause between each slice. This resulted in a field-of-view of 14.1 × 6.3 × 14.2 mm^3^, covered once every 42 seconds. During the single-slice ultrasensitive Doppler acquisition, the probe position was left to the discretion of the researcher, who looked for a coronal brain slice with the largest volume of hypoperfusion to image as the filament would be retracted.

#### Ultrasound localization microscopy

For each ULM experiment, 500 µL of clinically approved microbubble solution (SonoVue, Bracco) was injected in the tail vein to reconstruct CBFv maps. The ULM acquisition and processing procedure are described in Hingot et al.^12^ A washout period of 15 minutes elapsed before ultrasensitive Doppler measurements were resumed.

### Magnetic resonance imaging (group A and group B)

After uUS, Group A animals were transferred to a 7 T small-animal MR system (Bruker Biospin, Ettlingen, Germany) equipped with a quadrature head surface coil. Sequential T_2_-weighted images (multi-echo multi-slice TurboRARE) were acquired using repetition time (TR)/echo time ([TE]) = 2700/[11, 33, 55, 77] ms, RARE factor 2. Field-of-view (FOV) was set to 25.6 × 25.6 × 15.0 mm^3^, containing twenty-five contiguous 0.6 mm thick 256 × 256 coronal slices, resulting in a 100 × 100 × 600 μm^3^ voxel size. The timing of the MRI experiments in Group A are depicted in Figure 1.

The timing of the DSC-MRI experiments in group B are depicted in Figure 1. DSC-MRI experiments were performed on a 9.4 Tesla small-animal MR system (Varian, Palo Alto, CA) equipped with a Helmholtz volume coil (Ø 80 mm) and an inductively-coupled surface coil (Ø 25 mm) transmit-receive system. Prior to each DSC-MRI experiment, T_2_-weighted imaging was performed with multi-slice spin echo-planar imaging: TR/[TE] = 3000/[30, 50, 80, 190] ms, 33.75 × 33.75 ×15.00 mm^3^ FOV, twenty-five contiguous 0.6 mm thick coronal slices, and 224 × 224 matrix size, resulting in 150 × 150 × 600 μm^3^ voxel size. For DSC-MRI, 2 D multi-slice gradient-echo EPI was performed (TR/TE 164/13 ms, FOV 31.2 × 31.2 × 10.8 mm^3^; six contiguous 1.2 mm thick coronal slices containing a 64 × 64 data matrix with a 0.6 mm slice gap, 0.4875 mm^2^ in-plane resolution), combined with an intravenous injection of Gd-DTPA (Gadobutrol, Bayer Healthcare, Germany) (0.35 mmol/kg), injected at the 180th image. DSC-MRI experiments were separated by a contrast agent washout period of one hour. Three DSC-MRI scans were excluded due to technical issues.

### Image processing

#### uUS

All longitudinal ultrasensitive Doppler acquisitions were corrected for motion and coregistered using Advanced Normalization Tools (ANTs) software.^19^ For each subject, the time-average of the baseline 4 D ultrasensitive Doppler acquisition functioned as subject-specific reference image, to which each subsequent ultrasensitive Doppler acquisition was coregistered for analysis. Next, all coregistered ultrasensitive Doppler data were interpolated to a single common experimental timeline (temporal resolution of one minute) in a voxel-wise operation. ultrasensitive Doppler signal was linearly extrapolated to moments in the experimental timeline where data acquisition was not possible. This occurred for example during MCAO microsurgery and microbubble washout periods. Lastly, for each subject, the final whole-brain ultrasensitive Doppler acquisition preceding the MRI scan was manually co-registered to its corresponding T_2_-map using ITK-SnAP (v3.8.0)^20^ to facilitate alignment of lesion masks with uUS data. ULM images are acquired at the preceding ultrasensitive Doppler acquisition and do not need further registration.

#### DSC-MRI

MRI images were corrected for B_1_ inhomogeneity^21^ and brain extraction was performed with FSL’s Brain Extraction Tool.^22^ For each subject, the T_2_-weighted image with the shortest TE (i.e. least T_2_-weighting) was used as input for coregistration, which was performed with ANTs.^19^ Quantitative T_2_-maps were calculated by nonlinear monoexponential fitting of T_2_-weighted image data using the Levenberg-Marquardt algorithm. Maps of cerebral blood flow (CBF) were calculated by circular deconvolution of tissue concentration curves using a contralesional arterial reference curve,^23^ and CBV was calculated by numeric integration of the tissue concentration curve truncated at the 400th image. It is important to note that CBF represents a flow index, while ULM-derived CBFv represents actual flow velocity. Nevertheless, these two parameters are closely related.^24^

### Region-of-interest (ROI) analyses

Edematous tissue was semi-automatically segmented from T_2_-maps using in-house MATLAB code as described previously.^25^ The contralesional homologous area was obtained by co-registration of a subject’s T_2_-weighted image to a mirrored version of itself. These ROIs were then aligned to other modalities (uUS and ULM, or DSC-MRI, depending on the experimental group).

#### uUS

Since uUS-derived signal intensity is proportional to CBV, intensity values were related to internal control values. uUS relies on an endogenous source of contrast (i.e., the amount of red blood cells), therefore CBV can be expressed relative to baseline, assuming that red blood cell content and shear rates do not change over the course of the experiment. To achieve this, all ultrasensitive Doppler signal was divided with the median of the baseline signal in a voxel-wise operation, producing relative CBV (denoted rCBV_US_). For comparison with DSC-MRI (see below), CBV-weighted signal intensities were extracted from the ipsilesional ROI and expressed as a percentage of the signal from the contralesional ROI, denoted ‘normalized CBV’.

To exclude voxels that were not part of the cerebral vasculature, CBV-weighted signal was only extracted from voxels that exhibited sufficient variability over time (from baseline until the end of the experiment), which was achieved by voxel-wise calculation of the coefficient of variation and subsequently filtering voxels that did not exceed a heuristically derived threshold of 0.15. Lastly, ultrasensitive Doppler is prone to transient high-intensity flash artifacts. Values exceeding three times the mean signal in the ROI at a particular time were rejected to mitigate their contribution to the averaging process.

Absolute cerebral blood flow velocity (CBFv) values were extracted from ULM images. Only non-zero pixels were included in the averaging process.

#### DSC-MRI

Similar to uUS, for each subject lesional ROIs were overlaid on DSC-derived CBV and CBF image from every experimental time point to extract ipsi- and contralesional means. For DSC-MRI it was not possible to capture a baseline and relate it to subsequent measurements, since minute differences in injection efficiency (caused by variation in injection speed or catheter movement) frequently introduce erratic variation between time points, rendering intra-individual comparisons of raw CBV-weighted signal unreliable.

### Statistical analyses

To analyze ipsi- and contralesional differences in ultrasensitive Doppler relative to baseline (rCBV_US_) over time, linear mixed-model analysis was performed, using rCBV_US_ from ipsi- and contralesional ROIs during occlusion (halfway through at 45 minutes), and at one, two and three hours after tMCAO. ‘Subject’ (i.e. rat) was set as random effect. The model for CBFv data was identical but time points were baseline, occlusion, 0.5 h and 3 h post-recanalization.

The rCBV_US_ course from the single-slice ultrasensitive Doppler acquisition after recanalization was fitted, in a pixel-wise fashion, with a linear model, to estimate rCBV immediately after filament retraction. The fit yielded an intercept representing rCBV upon recanalization. The average intercept value was sampled from the ipsi- and contralesional ROI and compared in a paired t-test.

Linear mixed modeling was applied to compare raw CBF- and CBV-weighted signal readouts from DSC-MRI (denoted CBF_DSC_ and CBV_DSC_), testing for effects between ipsi- and contralesional ROIs over time. In order to account for inadvertent differences in injection efficiency at each time point (see above), ‘subject’ was nested in ‘time’, and treated as a random effect.

Lastly, to compare intra-hemispheric CBV-weighted signal differences between uUS and DSC-MRI, we expressed the mean ipsilesional signal intensity as a percentage of the contralesional signal intensity per measurement. The normalized CBV (i.e., normalized by a contralateral control value) was then compared between Group A and Group B over time in a linear mixed model, where ‘subject’ (rat) was set as random effect, and ‘modality’ (uUS or DSC-MRI) and ‘time’ were fixed effects.

Assumptions for parametric tests such as normality of residuals were checked at all times but no violations were encountered. Kenward-Roger corrections for small sample sizes were applied to the denominator degrees-of-freedom. Post-hoc tests were adjusted for multiplicity by Bonferroni’s method where necessary.

Statistical analyses were performed in R (v4.0.2)^26^ using packages *tidyverse,*^27^ *lme4*,^28^ and *emmeans*.^29^

## Results

### Profound drop of CBV during MCA occlusion, and incomplete restoration after recanalization

**Figure 2.**
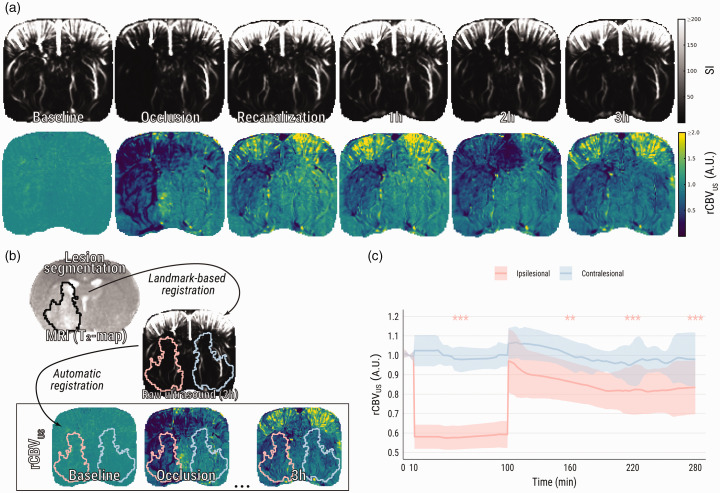
Ultrasensitive Doppler shows loss and subsequent incomplete restoration of CBV after tMCAO. (a) Representative images of raw ultrasensitive Doppler signal and relative CBV (rCBV_US_) over the course of an experiment. (b) Visual representation of the registration pipeline: MR images were co-registered with the final ultrasound images, while ultrasound images were automatically co-registered to one another. This pipeline facilitated alignment of vasogenic lesion masks from MRI (black outline on T_2_-map) to every previously recorded ultrasound image and (c) Group averages of relative CBV from ipsi- and contralesional ROIs. Asterisks indicate significant difference of ipsilesional relative CBV with respective to baseline. SI: signal intensity. **p < .01; ***p < .001.

tMCAO visibly induced ultrasensitive Doppler signal decrease and, analogously, rCBV_US_ loss within the ipsilesional hemisphere compared to baseline (0.5 ± 0.1, *p* < .0001) (Figure 2(a)). After withdrawal of the filament, ipsilesional rCBV_US_ restored to 0.88 ± 0.16 compared to baseline (*p* = .23). However, rCBV_US_ gradually declined thereafter, evident after one hour (0.81 ± 0.14, *p* = .003), two hours (0.78 ± 0.16, *p* < .0001), and three hours (0.80 ± 0.18, *p* < .0001). There was a significant interaction effect between ipsi- and contralesional ROIs over time, suggesting that time-dependent decreases in rCBV_US_ (compared with baseline) were not equal between ROIs: post-hoc testing indicated that, at three hours post-recanalization, the difference in rCBV_US_ with baseline was significantly larger in the ipsilesional ROI as compared to the contralesional ROI (*p* = .04).

### Temporary restoration of cerebral blood flow velocity after tMCAO

**Figure 3.**
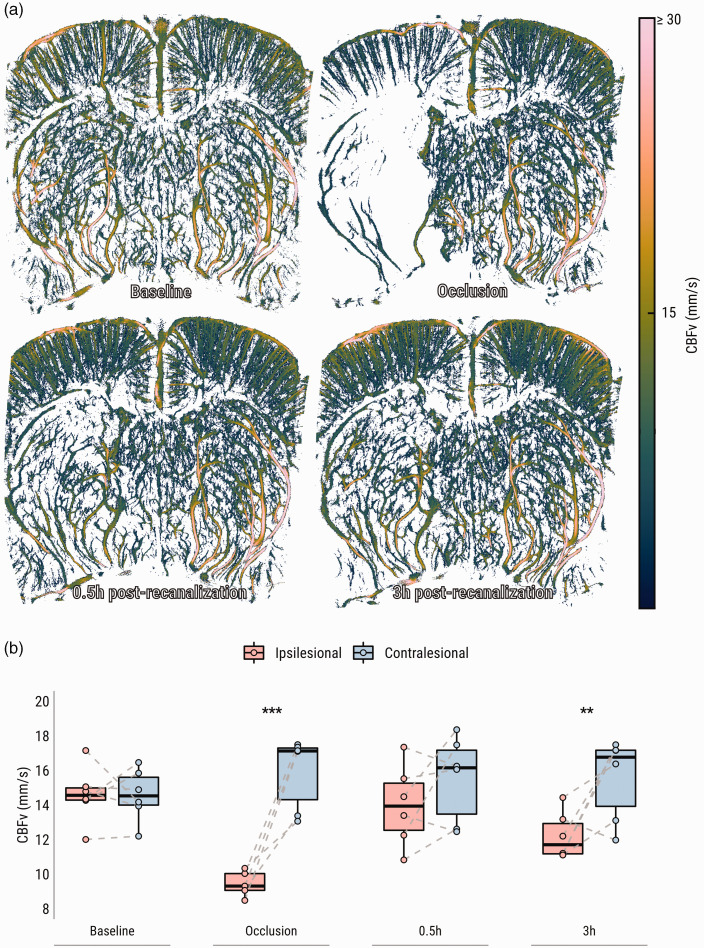
Ultrafast localization microscopy (ULM) shows loss and subsequent incomplete restoration of CBFv after tMCAO. (a) CBFv maps from a representative subject (Figure 2(a)) throughout the experiment and (b) Comparisons of CBFv group averages per hemisphere per time point. CBFv: cerebral blood flow velocity. **p < .01; ***p < .001.

Baseline values of CBFv were equal in the ipsi- and contralesional ROIs (14.6 ± 1.7 vs. 14.6 ± 1.5 mm/s). During occlusion, perfusion in ipsilesional pixels was often too low for detection (Figure 3(a), second row). In non-zero pixels, CBFv was nevertheless lower than in the contralesional area (Figure 3(b); 9.1 ± 1.1 vs. 15.9 ± 2.1 mm/s, *p* < .0001) and compared to baseline (*p* < .001). After recanalization, CBFv initially equalized between both hemispheres (14.0 ± 2.3 vs. 15.5 ± 2.5 mm/s), but ipsilesional CBFv was significantly reduced again compared to contralesional, at three hours after recanalization (Figure 3(b); 12.2 ± 1.4 vs. 15.5 ± 2.4 mm/s, *p* = .003).

### Recanalization induces a brief hyperperfusion response

**Figure 4.**
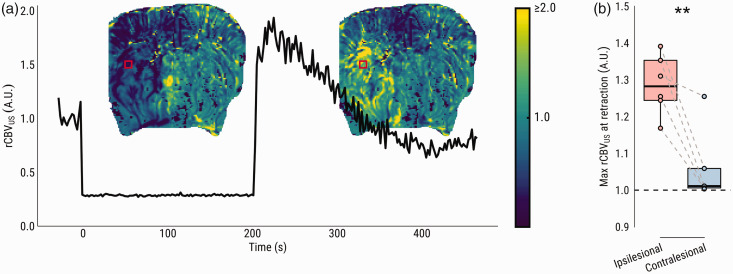
Single-slice ultrasensitive Doppler (SS-uD) shows immediate hyperperfusion and fast CBV decay after filament retraction. (a) rCBV during filament occlusion obtained by single-slice ultrasensitive Doppler from the same representative subject (Figures 2(a) and 3(a)). The line plot shows an example rCBV_US_ time series from the marked red square area, from baseline to occlusion, and ultimately the moment of filament retraction until the acquisition ends five minutes later. Maps of estimated rCBV_US_ (based on the intercept from a linear model fit) upon retraction are superimposed on the line plot, demonstrating an overshoot in the previously hypoperfused area and (b) Comparison of the average estimated rCBV upon filament retraction between ipsi- and contralesional ROI. **p < .01.

In single slice (2 D) acquisition mode, CBV could be tracked at a temporal resolution of 0.5 Hz, during MCAO and directly following retraction of the intraluminal filament (Figure 4(a)). Upon recanalization, an immediate rCBV_US_ overshoot occurred in the previously hypoperfused region (Figure 4(b)) (*p = *.007).

### DSC-MRI corroborates CBV changes measured with uUS after tMCAO, but not during MCA occlusion

**Figure 5.**
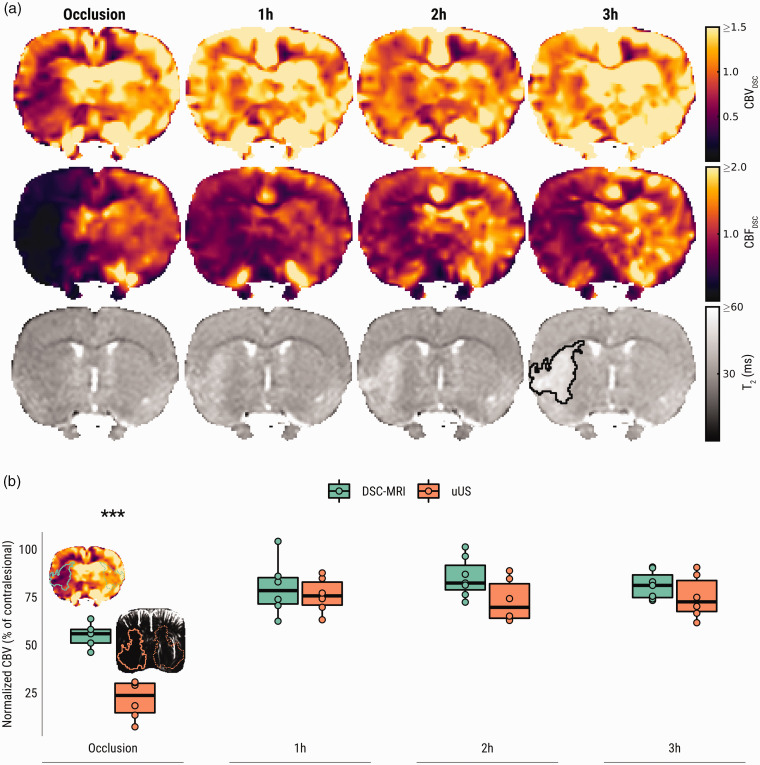
DSC-MRI shows loss and subsequent incomplete restoration of CBF and CBV after tMCAO, corroborating incomplete CBV reinstatement measured by uUS. (a) DSC-MRI-derived CBV (first row), CBF (second row) and T_2_-maps (third row) from a representative subject from Group B. CBV_DSC_ and CBF_DSC_ maps are displayed with a narrow intensity range to exemplify the hemispheric perfusion difference. The developing vasogenic edematous lesion is delineated in black on the T_2_-map recorded three hours post-recanalization and (b) Mean DSC-MRI and uUS-derived normalized ipsilesional CBV (as a percentage of contralesional values) at different time points during and after tMCAO. Representative images from the occlusion phase are shown with ipsi- and contralesional regions-of-interest (ROIs) overlaid. Each data point in this plot represents the average pixel intensity from the ipsilesional ROI (solid outline) expressed as a percentage of the contralesional ROI (dotted outline). ***p < .001.

Figure 5(a) shows representative examples of DSC-MRI-derived CBV and CBF maps, as well as T_2_ maps, during and after tMCAO. In agreement with our uUS findings, DSC-MRI-derived CBV-weighted signal was lowered in the ipsilesional ROI compared to the contralesional ROI, during occlusion (*p < *.0001), and one hour (*p = *.0008) and three hours after recanalization (*p = *.006) (Supplementary Figure 1a). Similarly, CBF-weighted signal was lower in the ipsilesional ROI during occlusion (*p* < .0001), one hour (*p* = .0005), two hours (*p* = .004) and three hours (*p* < .0001) post-recanalization (Supplementary Figure 1 b).

Mean ipsilesional CBV ratios (±standard deviation), compared to contralesional control values (i.e., normalized), was 0.56 ± 0.07 during occlusion, returning to 0.80 ± 0.15 one hour after recanalization, 0.85 ± 0.10 after two hours, and finally 0.81 ± 0.07 three hours after recanalization (Figure 5(b)). Similarly, mean normalized ipsilesional CBF ratios were 0.20 ± 0.08 of contralesional control values during occlusion, 0.66 ± 0.06 one hour after recanalization, 0.72 ± 0.11 after two hours, and finally 0.65 ± 0.11 three hours after recanalization (not shown).

Lastly, both uUS- and DSC-MRI derived measures of normalized ipsilesional CBV (as a ratio of contralesional values) were compared (Figure 5(b)). There was a significant main effect of modality (uUS vs. DSC-MRI) on the normalized CBV over time, however post-hoc testing showed this effect was only apparent during the occlusion phase of the experiment (*p < *.0001).

## Discussion

Here we compared CBV readouts using the emerging uUS technology with the established DSC-MRI technique in a dominant model of cerebral ischemia-reperfusion, i.e. filament-induced tMCAO, in rodents. After a 90-minute period of MCAO-induced hypoperfusion, uUS and ULM revealed restoration of CBV and CBFv, respectively, followed by a second persisting phase of moderate hypoperfusion, evident through comparison with baseline perfusion and contralateral control values. Filament retraction, concurrently with uUS acquisition, allowed instantaneous imaging of flow restoration, revealing a strong but short-lived CBV hyperperfusion response within the soon-to-be edematous lesion. Traditional DSC-MRI measurements corroborated these findings by showing comparable incomplete flow restoration after recanalization on CBF and CBV maps, although CBV deficiencies during occlusion were less pronounced compared to uUS.

There is a considerable amount of literature dedicated to elucidating causes and consequences of various (re)perfusion deficits, using different methods of measurement, in clinical and translational settings.^30^ Reperfusion dynamics are challenging to study due to their multifaceted nature and often fleeting responses to ischemic events, as demonstrated in the present analyses. Preclinically, a wide array of reperfusion responses have been reported within hours after stroke, ranging from hypoperfusion to hyperperfusion (or hyperemia). In the literature, variation in reperfusion status has been attributed to a wide array of factors such as spatial location, mode of recanalization (mechanical or thrombolytic), and vascular comorbidity.^30^

Progressive hypoperfusion was detected in the edematous lesion with uUS and DSC-MRI within hours following recanalization. While in agreement with earlier studies conducted with MRI^31^ or functional ultrasound^13^ in the same model, it conflicts with our previous experiments, where immediate hyperemic responses were detected in the post-ischemic lesion early after 90-minute tMCAO in rats.^5,25^ One possible explanation for absence of hyperperfusion early after recanalization in the current and other experiments^13,31–33^ is that data was collected while the animal was breathing freely under anesthesia, whereas in literature where acute hyperperfusion was reported, subjects (i.e., rats or cats) were mechanically ventilated.^5,25,34–36^ Compared to mechanically ventilated rats, free-breathing rats are chronically hypercapnic and acidemic, exhibiting fluctuating blood gas levels and low blood pressure, which has been shown to ultimately lead to larger infarct volumes and higher mortality.^37^ Another experiment in mechanically ventilated rats demonstrated that regional cerebral perfusion in post-ischemic tissue was sustained at control levels after 60-min tMCAO, although CO_2_-induced vascular reactivity was impaired.^38^ It is conceivable that the abnormal physiologic status caused by prolonged free breathing under anesthesia with volatile gas interacts with areas of ischemic injury and vasoparalysis to locally decrease blood flow. Post-ischemic blood vessels, already dilated and under metabolic distress, are further incapacitated by hypercapnic acidosis,^39^ which sets the stage for low vascular resistance and chronic hypoperfusion.^40^ As literature comparing hemodynamics after experimental AIS under free and assisted respiration is currently lacking, and while such a comparison was beyond the scope of our study, we speculate this experimental parameter can explain a considerable amount of variability in post-stroke reperfusion profiles in preclinical AIS literature.^30^

Despite the similarity in the pattern of changes in perfusion indices measured with uUS and DSC-MRI after recanalization, some discrepancy was noted when comparing normalized ipsilesional CBV ratios during occlusion, which were significantly lower when measured with uUS. While readouts from both techniques are related to CBV, they can be affected by processing steps or experimental factors, which can explain the observed difference. Regarding uUS, the ultrasensitive Doppler signal is preprocessed by a high-pass filter that rejects echoes from tissue or low-frequency movement such as respiration. However, as shown here with both ULM (Figure 3) and DSC-MRI (Figure 5), blood flow is very low during occlusion. Thus, echoes from red blood cells are effectively filtered out and CBV can be underestimated. Although we did not detect any differences between normalized CBV and CBFv shortly after recanalization, progressive decline in blood flow was evident in the edematous lesion (Figure 3), which may contribute to minor CBV underestimation. The unwanted rejection of slow flow can be mitigated by reducing the weighting of the filter, at the cost of allowing artifacts (such as respiratory movement) to enter the image.

Regarding DSC-MRI, while the relaxivity changes exploited here are linearly related to the vascular contrast agent (gadolinium) concentration,^41^ the interactions between relaxivity, vessel architecture and MRI acquisition parameters are highly complex.^42^ Contrast agent passage produces various amounts of signal intensity change for similar quantities of contrast agent depending on vessel size (micro- vs microvasculature) and pulse sequence (gradient echo (GE) vs spin echo (SE)). GE sequences are sensitive to both micro- and macrovasculature – though weighted towards the latter – while SE is mostly sensitive to microvasculature.^43^ Larger vessels tend to ‘bloom’ on GE acquisitions,^9^ making them seem larger than they actually are, hence they contribute more voxels to the averaging process. Since CBV is relatively higher in these vessels, the average CBV could be somewhat overestimated, explaining the slightly higher normalized ipsilesional CBV observed with DSC-MRI.

A second relevant consideration is that certain regions on CBV maps generated from DSC-MRI can also be artefactually flow-weighted, especially under ischemic conditions.^9^ CBV is calculated by voxel-wise numerical integration of the first-pass concentration-time curve, but extracting only the first passage is infeasible as the contrast agent recirculates immediately after injection. This effectively leads to summation of the initial concentration-time curve with successive arrivals. In regions where flow is delayed, there is a lower rate of contrast agent arrival. In areas of low flow, fewer passes of contrast agent are recorded – particularly if scan durations are too short – and CBV will be underestimated.^9^ In fact, the only method for truly accurate CBV measurements requires infinite scan durations, which is not a practical solution. These observations imply that under conditions of severe flow delay, CBV-weighted signal from both uUS and DSC-MRI need to be interpreted with caution.

There are some limitations to our study. For one, we could not apply uUS and DSC-MRI to investigate CBV in the same subject, as the craniotomized skull would produce unacceptable artifacts in gradient-echo MRI scans. Furthermore, as explained above, neither uUS nor DSC-MRI is likely to provide accurate CBV readouts in situations of very low flow. To further elucidate the size of the discrepancy between these relative measures of CBV, additional readouts of absolute CBV would have to be collected. One technique capable of quantifying absolute CBV is gold-standard positron emission tomography combined with ^11^CO inhalation. However, we were unable to conduct these experiments due to the prohibitively expensive nature of on-site cyclotrons, which our institutions were lacking.

In conclusion, the present work demonstrates distinctive CBV and CBFv responses to filament-induced ischemia-reperfusion with ultrafast ultrasound through a partially craniotomized skull in rats. CBV and CBFv were incompletely restored, despite an initial transient hyperperfusion response, but this may be related to a free-breathing anesthesia regime. Results from nearly identical DSC-MRI experiments corroborated the incomplete flow restoration, however differences in normalized CBV ratios during MCA occlusion could be due to differences in underlying signal mechanisms and processing algorithms.

## Supplemental Material

sj-pdf-1-jcb-10.1177_0271678X231220698 - Supplemental material for Dynamics of cerebral blood volume during and after middle cerebral artery occlusion in rats – Comparison between ultrafast ultrasound and dynamic susceptibility contrast-enhanced MRI measurements Click here for additional data file. Supplemental material, sj-pdf-1-jcb-10.1177_0271678X231220698 for Dynamics of cerebral blood volume during and after middle cerebral artery occlusion in rats – Comparison between ultrafast ultrasound and dynamic susceptibility contrast-enhanced MRI measurements by Bart AA Franx, Florent Lebrun, Lois Chin Joe Kie, Thomas Deffieux, Denis Vivien, Thomas Bonnard, Rick M Dijkhuizen and on behalf of the CONTRAST consortium in Journal of Cerebral Blood Flow & Metabolism
