# Supplementary material for: Dynamics of cerebral blood volume during and after middle cerebral artery occlusion in rats – Comparison between ultrafast ultrasound and dynamic susceptibility contrast-enhanced MRI measurements
Source: J Cereb Blood Flow Metab. 2023 Dec 21;44(3):333–44. doi: 10.1177/0271678X231220698 (PMC10870967; doi:10.1177/0271678X231220698)
Supplement: sj-pdf-1-jcb-10.1177_0271678X231220698 - Supplemental material for Dynamics of cerebral blood volume during and after middle cerebral artery occlusion in rats – Comparison between ultrafast ultrasound and dynamic susceptibility contrast-enhanced MRI measurements [file sj-pdf-1-jcb-10.1177_0271678X231220698.pdf]

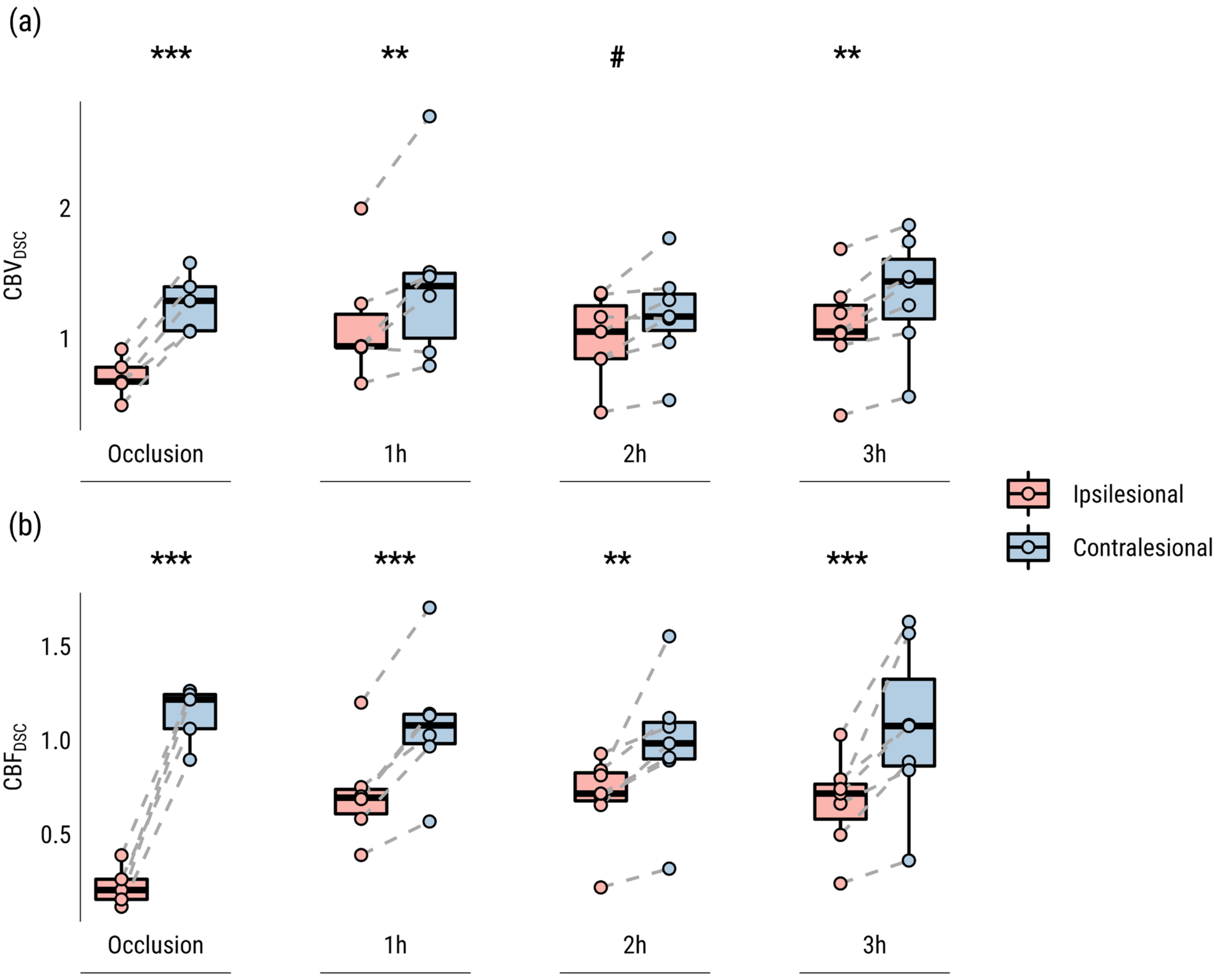

Supplementary Figure 1. DSC-MRI demonstrates hypoperfusion during and after MCA occlusion. **(a)** Lack of perfusion in the ipsilesional hemisphere as shown by raw CBV-weighted signal intensity and (**b)** CBF-weighted signal intensity. $^{\#}p < .10$; $^{**}p < .01$; $^{***}p < .001$.
